# Antibacterial Mechanism of 3-Carene against the Meat Spoilage Bacterium *Pseudomonas lundensis* and Its Application in Pork during Refrigerated Storage

**DOI:** 10.3390/foods11010092

**Published:** 2021-12-30

**Authors:** Zhiling Tang, Haiming Chen, Ming Zhang, Zhuye Fan, Qiuping Zhong, Weijun Chen, Yong-Huan Yun, Wenxue Chen

**Affiliations:** 1 College of Food Sciences & Engineering, Hainan University, 58 People Road, Haikou 570228, China; 19085231210031@hainanu.edu.cn (Z.T.); 992984@hainanu.edu.cn (H.C.); m.zhang@hainanu.edu.cn (M.Z.); 20170881310157@hainanu.edu.cn (Z.F.); 990511@hainanu.edu.cn (Q.Z.); chenwj@hainanu.edu.cn (W.C.); yunyonghuan@hainanu.edu.cn (Y.-H.Y.); 2 School of Maritime, Hainan Vocational University of Science and Technology, 18 Qiongshan Avenue, Haikou 571126, China

**Keywords:** *Pseudomonas lundensis*, 3-carene, preservatives, membrane damage

## Abstract

*Pseudomonas lundensis* is the main bacterium responsible for meat spoilage and its control is of great significance. 3-Carene, a natural monoterpene, has been proved to possess antimicrobial activities. This study aimed to investigate the antibacterial activity and mechanism of 3-carene against the meat spoilage bacterium *P. lundensis*, and explore its application on pork. After 3-carene treatment, cellular structural changes were observed. Cell walls and membranes were destroyed, resulting in the leakage of alkaline phosphatase and cellular contents. The decreased activity of Ca^2+^-Mg^2+^-ATPase and Na^+^-K^+^-ATPase showed the imbalance of intracellular ions. Subsequently, adenosine triphosphate (ATP) content and oxidative respiratory metabolism characteristics indicated that 3-carene inhibited the metabolism of the tricarboxylic acid cycle in *P. lundensis*. The results of binding 3-carene with the vital proteins (MurA, OmpW, and AtpD) related to the formation of the cell wall, the composition of the cell membrane, and the synthesis of ATP further suggested that 3-carene possibly affected the normal function of those proteins. In addition, the growth of *P. lundensis* and increase in pH were inhibited in pork during the 5 days of cold storage after the samples were pre-treated with 3-carene. These results show the anti-*P. lundensis* activity and mechanism of 3-carene, and its potential use in meat preservation under refrigerated conditions.

## 1. Introduction

Meat spoilage, which causes production losses as high as 40%, is of widespread concern in the meat industry [1]. Due to various nutrients, meat is an ideal environment for the growth and reproduction of meat spoilage bacteria and common foodborne pathogens. Refrigeration is usually the most common preservative method of meat and meat products [2]. However, the combination of a long storage time and a low temperature creates a selective advantage for psychrotrophic bacteria, especially *Pseudomonas* spp., which plays a major role in limiting the shelf life of fresh foods stored at cold temperatures [3]. *Pseudomonas lundensis*, a Gram-negative bacterium, is one of the dominant spoilage bacteria in refrigerated meat [4,5]. *P. lundensis* is able to produce key volatile organic compounds (VOCs) during its growth on beef paste, such as methyl-2-butenoic acid, with its rancid, pungent odor [6]. It had strong proteolytic activity when causing the spoilage of chilled pork [7], due to the production of a large amount of extracellular protease and lipase [5]. In order to inhibit the reproduction of bacteria and extend the shelf life of food products, essential oils (EOs), extracted from plants, or their active ingredients, are increasingly being used as antimicrobial agents in food preservation, because of their low toxicity to non-target species and greater safety compared to synthetic antimicrobial agents [8].

*Piper nigrum* L., most commonly known as pepper, is one of the most consumed spices and is widely used in food condiments. *Piper nigrum* and its bioactive compounds have been reported to possess antioxidant, anti-inflammatory, and antimicrobial properties [9]. Our previous studies identified that 3, 7, 7-trimethyl-bicyclo (4, 1, 0) hept-3-ene (3-carene) is the volatile compound with the highest relative content in black pepper and white pepper, accounting for 31.33% and 27.02%, respectively [10]. 3-Carene is a kind of monoterpenoid and has been proved to be one of the most abundant compounds in *Piper nigrum* oil [11]. According to GB 2760-2014 (Chinese standard), 3-carene can be applied in food as a spice. It has been used as a raw material in perfume, cosmetics, and spices and has anti-inflammatory, anti-fungal, and sedative effects [12,13]. Huynh et al. [14] discovered that 3-carene was a major volatile compound of four pepper fruit EOs extracted by four extraction methods, and two types of EOs with a high content of 3-carene had antibacterial activity against *Escherichia coli* and *Bacillus subtilis*. Moreover, 3-carene strongly inhibited biofilm formation in *Staphylococcus aureus* and the minimum inhibitory concentration (MIC) was 10.39 mg/mL [15]. Although 3-carene has been shown to be effective against bacteria, there are few reports on its antibacterial action and mechanism, especially regarding meat spoilage bacteria. Our previous research discovered that the adverse changes in the cell wall and membrane, energy synthesis, and DNA structure of *Brochothrix thermosphacta* and *Pseudomonas fluorescens* were caused by 3-carene [16].

However, at present, no studies on the antibacterial mechanism of 3-carene against *P. lundensis* and its preservation effect on meat have been reported. Our research addressed this gap and the interaction between 3-carene and specific targets was explored. In this study, combined with the in vitro studies and the molecular docking of 3-carene with UDP-N-acetylglucosamine-1-carboxy vinyl transferase (MurA), outer membrane protein W (OmpW) and F1F0 ATP synthase F1 sector subunit β (AtpD), the antibacterial activity and mechanism of 3-carene were explored and the potential target proteins to which 3-carene binds were predicted. Furthermore, the antibacterial effects of 3-carene on pork samples at 4 °C were assessed.

## 2. Materials and Methods

### 2.1. Bacterial Strains and Chemicals

*P. lundensis* ATCC 49,968 was purchased from the BeNa Culture Collection (Beijing, China). Before all experiments, the strain was revitalized at 28 °C in nutrient agar.

The (+)-3-Carene (≥90.0%) was purchased from Tokyo Chemical Industry Co., Ltd. (Tokyo, Japan). Rhodamine 123 was purchased from Shanghai Yuanye Bio-Technology Co., Ltd. (Shanghai, China). Coomassie Brilliant Blue G250 was purchased from Aladdin Co., Ltd. (Shanghai, China). The ATPase, adenosine triphosphate (ATP), bicinchoninic acid (BCA), and alkaline phosphatase (AKP) assay kits were purchased from Nanjing JianCheng Bioengineering Institute (Nanjing, China). All other chemicals were analytical grade.

### 2.2. Determination of MIC

The MIC of 3-carene against *P. lundensis* was tested using an agar dilution method [17]. Using ethanol (20%, *v/v*) as solvent, the 3-carene concentrations of 300, 250, 200, 150, 100 and 50 mL/L were obtained. Trials were performed in nutrient agar. All prepared test compounds were dissolved in agar at a ratio of 1:9 to obtain final concentrations of 30, 25, 20, 15, 10, and 5 mL/L, respectively. When agar solidified, 100 μL suspensions containing 10^6^–10^7^ CFU/mL of bacteria were inoculated. Then, those samples were incubated at 28 °C for 24 h. The blank (bacterial suspensions and sterile water) and negative control (bacterial suspensions and ethanol with a final concentration of 2%) groups were used to observe normal bacterial growth and to exclude the effect of 2% ethanol on bacterial growth, respectively.

### 2.3. Time–Kill Assay

The time–kill curves of 3-carene against *P. lundensis* were evaluated according to a flat colony-counting method [18]. 3-Carene solution prepared according to the method in Section 2.2 was added into the bacterial suspension with the concentration of 8–9 log CFU/mL to obtain final concentrations (1 × MIC and 2 × MIC ). At different times (0, 4, 8, 12, and 24 h), 1 mL of sample was drawn from each tube and diluted into 9 mL of saline solution (NaCl 0.9%) in a gradient 10 times to obtain appropriate bacterial concentration. Afterwards, 100 μL of each dilution was coated on plates and incubated for 48 h.

### 2.4. Cell Size Assay

The bacterial cell size distributions were carried out according to the method of Kwiatkowski et al. [19]. The different concentrations of 3-carene (1 × MIC, 2 × MIC) were added to bacterial cells cultured to the logarithmic phase. After incubation at 28 °C for 8 h, the cells were washed with phosphate-buffered solution (PBS, 0.01 M, pH = 7.2) three times before centrifugation (H1850R, Xiangyi Centrifuge Instrument Co., Ltd., Changsha, Hunan, China). Finally, bacterial suspensions were measured on a laser particle sizer (Zetasizer Nano ZS90, Malvern Instrument Ltd., Malvern, UK)

### 2.5. Morphological Changes

According to the method described in the literature [20], scanning electron microscopy (SEM) was used to observe the morphological changes in cells exposed to 3-carene at 1 × MIC and 2 × MIC. The samples were incubated at 28 °C for 12 and 24 h, centrifuged (1500× *g*, 10 min), and washed three times by PBS. Then, the bacteria were fixed with 2.5% (*v/v*) glutaraldehyde (4 °C, 4 h) and washed with PBS. The cells were dehydrated successively in a series of ethanol gradients (20%, 40%, 60%, 80%, and 100%). Finally, the freeze-dried samples were sputter-coated with gold under vacuum and photographed by SEM (Verios G4 UC, Thermo Fisher Scientific Inc., Waltham, MA, USA).

### 2.6. Determination of Cell Wall Integrity

Bacteria were treated by 3-carene at 1 × MIC and 2 × MIC in culture medium at 28 °C. After 0, 4, 8, 12, and 24 h, the supernatants were collected. The cellular AKP activity was determined using an AKP kit with a microplate reader (SP-Max3500FL, Shanghai Flash Spectrum Biotechnology Co., Ltd., Shanghai, China).

### 2.7. Ions Release Assay

After incubation with 3-carene at 1 × MIC and 2 × MIC for 0, 30, 60, 120, and 240 min, respectively, bacterial suspensions were centrifuged at 10,000× *g* at 4 °C for 10 min. Then, all supernatants were filtered through a 0.22 μm membrane, and the concentrations of free K^+^ and Mg^2+^ leaking in supernatants were measured on an atomic absorption spectrometer (TAS-990 Super AFG, Beijing Purkinje General Instrument Co., Ltd., Beijing, China).

### 2.8. Protein and Nucleic Acid Release Assay

The integrity of the cell membrane was evaluated by measuring the release of proteins and cell nucleic acids into the cell suspension. The samples were prepared according to the same steps as mentioned in the above section. The absorption was measured at 260 nm on a microplate reader and the determination of protein content was measured by Coomassie Brilliant Blue.

### 2.9. ATPase Activity and ATP Content

After incubation with 3-carene, the cells were washed three times with sterile saline (NaCl, 9%) and resuspended. Then, cells were treated with ultrasonic processing in an ice bath (550 W, 5 s interval). The supernatant was obtained by centrifugation and stored at 4 °C. The intracellular protein concentration was measured by the BCA protein assay kit with a microplate reader (Synergy LX, Biotek Instruments Inc., Winooski, VT, USA) at 562 nm. Then, according to the manufacturer’s instructions for the ATPase and ATP assay kit, the samples were transferred to a 96-well microtiter plate and determined by a microplate reader.

### 2.10. Oxidative Respiratory Metabolism Assay

Oxidative respiratory metabolism was measured by dissolved oxygen (DO) according to the method reported by Lin et al. [21]. A total of 1 mL of bacterial suspension was mixed with 3.6 mL PBS and 0.4 mL 1% glucose solution. After treatment in air for 5 min, the amount of dissolved oxygen in the initial solution was measured using a liquid-phase oxygen measurement system (Qxytherm+R, Hansatech Instruments Ltd., Norfolk, UK), and the initial respiratory rate, marked R_0_, was the difference in DO values per minute. Then, the respiratory rate of different mixtures which contained, respectively, three inhibitors (iodoacetate, malonic acid, sodium phosphate) and 3-carene with a final concentration of 1 × MIC was named R_1_, and the respiratory rate after adding a mixture of 3-carene and each inhibitor was R_2_. The inhibition rate and superpose rate were calculated from two following equations:

$$\mathrm{Inhibition}\;\mathrm{rate}\;\left(I_{R}\right)=\frac{R_{0}-R_{1}}{R_{0}}\times 100\%$$

$$\mathrm{Superpose}\;\mathrm{rate}\;\left(S_{R}\right)=\frac{R_{1-}R_{2}}{R_{1}}\times 100\%$$

### 2.11. Molecular Docking of 3-Carene with Three Target Proteins (MurA, OmpW and AtpD)

Because of the unavailability of MurA, OmpW, and AtpD crystal structures of *P. lundensis* in the Protein Data Bank (PDB, https://www.rcsb.org/, accessed on 19 June 2021), the construction of the three proteins’ three-dimensional (3D) structures was performed using homology modeling. The amino acid sequence acquired from the National Center for Biotechnology Information (NCBI, https://www.ncbi.nlm.nih.gov/, accessed on 9 June 2021) was submitted to SWISS-MODEL (https://swissmodel.expasy.org/, accessed on 19 June 2021) for sequence alignment and homology modeling, in order to obtain a high sequence identity (>30%) template. On the basis of the coordinates of the template’s original ligand, the active sites were found in Discovery Studio 4.5. Then, the 3D structures of molecules including fosfomycin (inhibitor), (hydroxyethyloxy) tri (ethyloxy) octane (original ligand), adenosine diphosphate (ADP, substrate), and 3-carene were obtained from the PubChem database. AutoDock Tools was used to analyze the interaction between small-molecule ligands and protein active sites and to calculate the binding energy of the protein–ligand complex. The docking pose and binding energy of the protein–3-carene complex were compared with that of each protein, bound to an inhibitor, original ligand or substrate.

### 2.12. Application of 3-Carene as an Antimicrobial Marinade in Pork

This assay was conducted as the method of Maragkoudakis et al. [22]. Fresh pork tenderloin was obtained from a local market (Haikou, China) and kept in an incubator (4 °C) before the experiment. Each pork sample (5 g) was dipped into 2% ethanol, 1 × MIC, and 2 × MIC 3-carene for 10 min, respectively, then was inoculated with *P. lundensis* (about 7.0 log CFU/g). After storage at 4 °C for 1, 2, 3, 4, and 5 days, the stored samples were crushed and diluted to the appropriate concentration, in order to be inoculated on Pseudomonas Agar Base with CFC supplement. Other samples were further analyzed for changes in pH by using a pH meter (Mettler Toledo Co., Ltd., Shanghai, China) with an accuracy of 0.01. Citric acid monohydrate and sodium hydroxide solution (pH = 5.45, 20 °C) and mixed phosphate solution (pH = 6.88, 20 °C) were used as calibration buffers.

### 2.13. Statistical Analysis

All the experiments were carried out in triplicate. Statistical analysis was performed using SPSS version 13.0 statistical software (SPSS Inc., Chicago, IL, USA). Graphs were created with Origin software (Origin Lab Co., Pro.8.0, Northampton, MA, USA). The differences were considered significant when *p* < 0.05, and *p* < 0.01 was used to indicate a greater significance.

## 3. Results and Discussion

### 3.1. Antibacterial Activities Analysis

As shown in Table 1, minimum inhibitory concentration (MIC) values reveal that 3-carene could inhibit the activity of *P. lundensis* at 25 mL/L (21.65 mg/mL). In addition, as the solvent of 3-carene, 2% ethanol did not affect the growth of *P. lundensis.*

**Figure 1:**
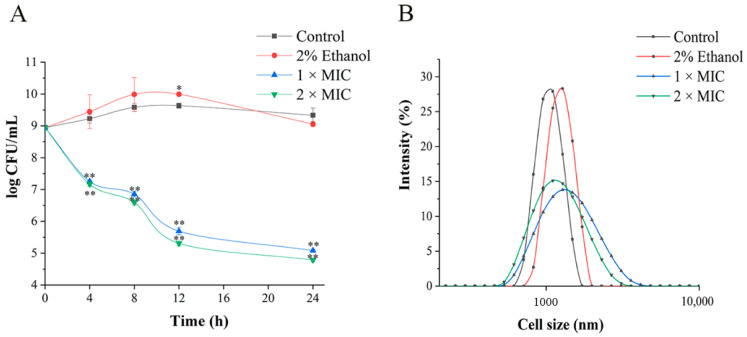
Time–kill curves analysis (**A**). The distribution of cell size (**B**). The asterisks indicate statistically significant differences compared with the control (* *p* < 0.05; ** *p* < 0.01).

To verify the antibacterial effect of 3-carene against *P. lundensis* and its rapidity and duration, the time–kill curves of bacteria cultured with sterile water, 2% ethanol and 3-carene (1 × MIC, 2 × MIC) were plotted, respectively. According to the Clinical and Laboratory Standards Institute (CLSI) protocols, the point of differentiation between bactericidal and bacteriostatic activity is a decrease in colony number by 3 log CFU/mL or more [23]. The results of the time–kill curves (Figure 1A) indicate that 3-carene was rapidly bactericidal against *P. lundensis* at 1 × MIC and 2 × MIC within 12 h. After treatment with 1 × MIC and 2 × MIC 3-carene for 24 h, the bacterial population decreased drastically from 8.94 to 5.08 and 4.78 log CFU/mL, respectively.

### 3.2. Destruction Effect of 3-Carene on the Structure of P. lundensis

As observed in Figure 1B, the size distribution of the bacteria in the control group was 0.7–1.5 μm, and in the ethanol-treated group was 0.8–1.7 μm. However, the size distributions outside the range of the control group after treatment with 3-carene (1 × MIC and 2 × MIC) for 8 h were extended to 0.4–4 μm and 0.5–3 μm, respectively. The results concerning cell size revealed that the range of cell distribution was expanded under 3-carene treatment, indicating that the cell aggregation was more severe.

**Figure 2:**
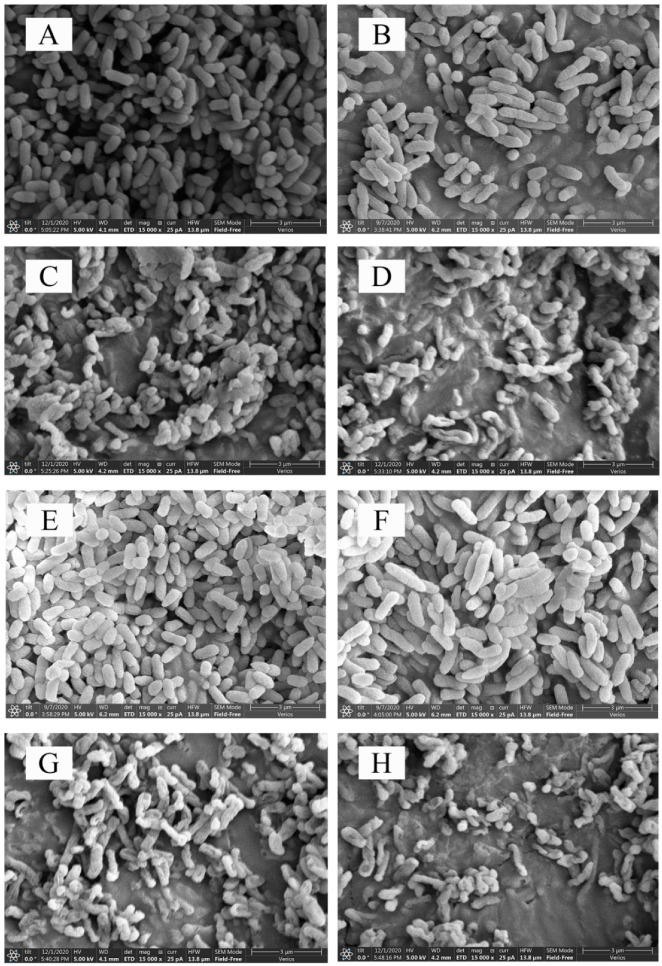
Scanning electron microphotographs (SEM) of *P. lundensis*. (**A**) *P. lundensis* untreated for 12 h, (**B**) treated with 2% ethanol for 12 h, (**C**) treated with 3-carene at 1 × MIC for 12 h, (**D**) treated with 3-carene at 2 × MIC for 12 h, (**E**) untreated for 24 h, (**F**) treated with 2% ethanol for 24 h, (**G**) treated with 3-carene at 1 × MIC for 24 h, and (**H**) treated with 3-carene at 2 × MIC for 24 h.

SEM was used to observe the overall structure of cells treated with 3-carene at different concentrations after 12 and 24 h. Compared with the control group (Figure 2A,E) and the ethanol-treated group (Figure 2B,F), which had an intact and smooth surface, uniform size, and typical characteristics of native cells, the cells after 3-carene treatment for 12 h (Figure 2C,D) showed obvious pits and an irregular shape. Most of the bacteria were withered and stacked together, as shown in Figure 2D. These phenomena were caused by the destruction of the cell wall and membrane. After 24 h of treatment with 3-carene (Figure 2G,H), the bacterial cells were obviously shriveled because the cytoplasmic content flowed out and the integrity of the cell membrane was completely destroyed. Furthermore, there were large holes on the surface of cells. The results in Figure 2C,D,G,H show that the damage to the cells was enhanced with increasing doses of 3-carene and treatment time. Based on the different destruction states of cells, it was speculated that the damage to the cells without complete disintegration after 24 h of treatment might be the reason for cell death [24].

The SEM observation further exposed the acute damage caused by 3-carene to the cell structure. Many researchers have reported that the direct damage of EOs and their derivatives to the microbial cell membrane is the main reason for their antibacterial activity, which can be proved in the cell wall destruction, cytoplasmic membrane damage, and leakage of intracellular substances [20]. Similarly, Dai et al. [25] found that *Litsea cubeba* EO destroyed the bacterial wall and membrane, and caused irreversible damage to the cell structure of *E. coli* O157:H7. Therefore, we inferred that 3-carene could inhibit bacteria by targeting the cell wall and membrane.

### 3.3. Cell Wall Damage Analysis

**Figure 3:**
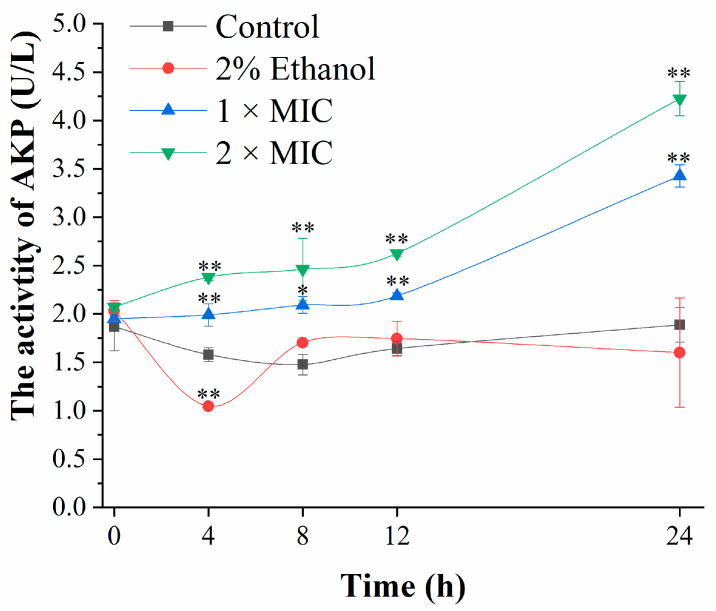
Alkaline phosphatase (AKP) activity of bacteria before and after 3-carene treatment. The asterisks indicate statistically significant differences compared with the control (* p < 0.05; ** *p* < 0.01).

Alkaline phosphatase (AKP) exists between the cell wall and the cell membrane and under normal circumstances is not secreted into the extracellular environment. Once enzyme activity was detected in the extracellular environment, the cell wall had been damaged [26]. The AKP activity of the bacteria in the control group and the ethanol-treated group had no obvious change (Figure 3), while the AKP activity in the 3-carene-treated group increased significantly with the concentration of 3-carene and incubation time. In general, the AKP activity of the 3-carene-treated group was always higher than that of the control. After incubation with 3-carene (1 and 2 × MIC) for 24 h, the AKP values were 1.81 and 2.24 times higher than that of the control, indicating that 3-carene caused irreversible damage to the cell wall. It has been proven that the terpenoids with strong fat solubility can dissolve the cell wall after interaction with bacteria [27].

### 3.4. Cell Membrane Damage Caused by 3-Carene

**Figure 4:**
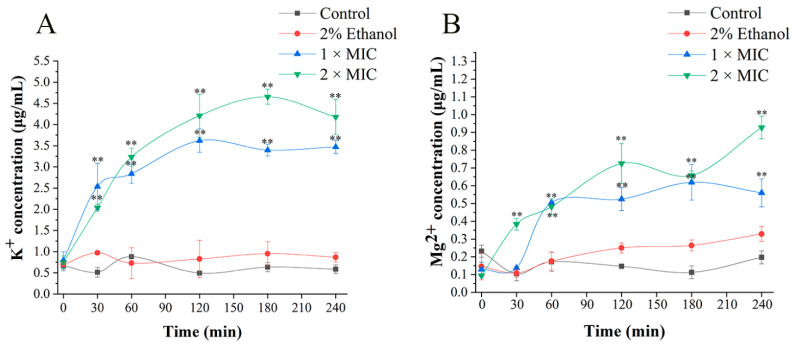
Effect of 3-carene on the concentration of extracellular K^+^ (**A**) and Mg^2+^ (**B**). The asterisks indicate statistically significant differences compared with the control (** *p* < 0.01).

Once cell damage occurs, the outflow of K^+^ and cell contents is considered a typical indicator [28,29]. In addition, Mg^2+^, as an important ion in cells, relates to the activity of Ca^2+^ -Mg^2+^-ATPase [30]. Therefore, K^+^ and Mg^2+^ were selected to evaluate the damage of the cell membrane. The leakage of K^+^ and Mg^2+^ caused by 3-carene is shown in Figure 4. After treatment with 3-carene, the concentrations of K^+^ and Mg^2+^ were both significantly higher than that of the control group, but there were some differences in trend. As observed in Figure 4A, the release of K^+^ sharply increased by 0.058 and 0.043 μg/mL per minute at 1 × MIC and 2 × MIC over 30 min and fluctuated between 3.39 and 3.62 μg/mL at 1 × MIC and between 4.17 and 4.65 μg/mL at 2 × MIC after 120 min, which maintained a relatively stable state. As for the release of Mg^2+^ (Figure 4B), with the treatment of 1 × MIC 3-carene, from 30 to 60 min, the Mg^2+^ concentration increased by 0.012 μg/mL per minute, then tended to be stable. However, the Mg^2+^ concentration showed an upward tendency over 240 min at 2 × MIC until it reached a maximum value of 0.928 μg/mL. During 3-carene treatment for 4 h, K^+^ and Mg^2+^ outflowed rapidly, and there was an imbalance of ions inside and outside the cell membrane. This indicates that the permeability of the cell membrane was changed in a short period of time, leading to the leakage of intracellular ions.

**Figure 5:**
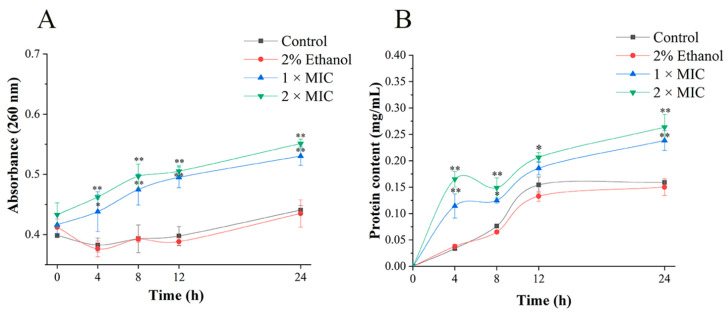
Effect of 3-carene on the release of extracellular nucleic acid (**A**) and protein (**B**). The asterisks indicate statistically significant differences compared with the control (* p < 0.05; ** *p* < 0.01).

According to Bajpai et al. [31], the membrane integrity could be reflected by cell leakage markers, including absorbance at 260 nm for the content of nucleic acids and protein. Figure 5 shows the results of the protein and nucleic acid release of *P. lundensis* after 3-carene treatment. With the increase in treatment time, the OD260 value of the treatment group gradually increased and was always higher than that of the control group (Figure 5A), especially at 12 h. Compared with the control, the OD260 value of cell suspensions treated with 1 × MIC and 2 × MIC 3-carene increased by 24% and 27% at 12 h. The extracellular protein contents of the treatment group increased to 0.29 and 0.26 mg/mL, respectively (Figure 5B).

We observed that the cell wall and cell membrane were injured along with the outflow of ions (K^+^ and Mg^2+^) and macromolecules (protein and nucleic acid), which increased in a time- and dose-dependent manner and was also consistent with the results of SEM analysis. Many other antibacterial agents exhibit similar actions. For example, Moghimi et al. [32] reported that nanoemulsions of *Thymus daenensis* EO contributed to potassium, protein, and nucleic acid leakage from the cells.

### 3.5. ATPase (Ca^2+^-Mg^2+^-ATPase, Na^+^-K^+^-ATPase) Activity and ATP Content

**Figure 6:**
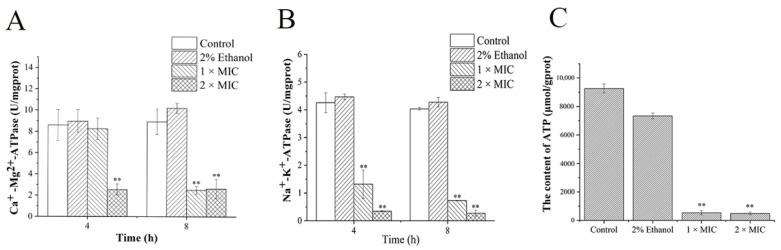
Effect of 3-carene on Ca^2+^-Mg^2+^-ATPase activity (**A**), Na+-K+-ATPase activity (**B**) and adenosine triphosphate (ATP) content (**C**). The asterisks indicate statistically significant differences compared with the control (** *p* < 0.01).

Ca^2+^-Mg^2+^-ATPase is a calcium pump on the cell membrane, which can hydrolyze ATP to pump intracellular Ca^2+^ out of the cell in order to maintain the relatively low intracellular Ca^2+^ concentrations [33]. Na^+^-K^+^-ATPase, existing in the cell membrane, contributes to the exchange of intracellular Na^+^ for extracellular K^+^ against the concentration gradient [27]. Figure 6A and B show that the activities of Na^+^-K^+^-ATPase and Ca^2+^-Mg^2+^-ATPase decreased after treatment with 3-carene for 4 and 8 h. The effect of 2 × MIC 3-carene on the activity of Ca^2+^-Mg^2+^-ATPase was first shown at 4 h (Figure 6A) and the activity decreased by 70.25%, compared with the control group. After treatment with 1 × MIC 3-carene for 8 h, the activity of Ca^2+^-Mg^2+^-ATPase decreased by 72.12%. At 4 and 8 h, Na^+^-K^+^-ATPase of 1 × MIC group declined acutely by 68.90% and 81.82%, respectively, while the Na^+^-K^+^-ATPase content of the 2 × MIC group decreased by 91.90% and 93.13%, respectively (Figure 6B). The decrease in the level of ATPase in *P. lundensis* treated with 3-carene indicated that 3-carene destroyed the activity of ATPase, affected the metabolic activity of the bacteria, and destroyed the cation balance in cells. It was also reported that the low activity of ATPase could lead to impeded respiratory metabolism [34], which corresponds to the oxidative respiratory metabolism analysis in Section 3.6.

After treatment for 4 h, the intracellular ATP content (Figure 6C) was also affected by 3-carene and decreased rapidly. The decrease in ATP content was possibly due to the excessive consumption of ATP caused by excessive apoptosis [35] or the decrease in MP, which is central to cellular proliferation and provides the essential driving force for ATP synthesis [36,37]. At 4 h, there were approximately 10^7^ living cells in the 3-carene-treated group, but the ATP content decreased significantly. Therefore, we speculated that the second reason was consistent and we analyzed the interaction between 3-carene and AtpD to verify the conjecture.

### 3.6. Oxidative Respiratory Metabolism Characteristics

There are three pathways of glucose decomposition, including the Embden–Meyerhof–Parnas pathway (EMP), tricarboxylic acid cycle (TCA), and hexose monophosphate pathway (HMP). Iodoacetic acid, malonic acid, and trisodium phosphate are the corresponding typical respiratory inhibitors. The inhibition rates of the three different typical respiratory inhibitors and 3-carene on *P. lundensis* were 81.12%, 40.02%, 29.87%, and 32.93%, respectively (Table 2). As shown in Table 3, the lowest superposition rate (22.13%) emerged in the group of 3-carene and a TCA inhibitor, which indicated that 3-carene had the highest inhibitory probability on TCA. The antibacterial mechanism of respiration metabolism has also been studied in many plant substances [38]. Our study confirmed that the TCA pathway was regulated by 3-carene.

### 3.7. Molecular Docking

According to the above analysis of *in vitro* experiments, we found that the cell structure was destroyed and cell metabolism was inhibited after 3-carene treatment. In this regard, the protective barrier for bacteria is the cell wall and membrane. Therefore, the proteins or enzymes that are in charge of the integrity or formation of the cell membrane and wall may be potential targets of antibacterial agents. The MurA, a recognized antibiotic target, is involved in the first step of bacterial cell wall biosynthesis [39]. Once MurA is inhibited, cells are triggered to break down [40]. OmpW is a β-barrel-shaped structural protein and one of the main components in the outer membrane of Gram-negative bacteria [41] and has been studied as a new drug target for *Acinetobacter baumannii*. OmpW has been proved to participate in the transport of hydrophobic or aromatic molecules through the outer membrane [42]. In addition, cell metabolism is related to ATP level. The AtpD hosts most of the catalytic sites and is responsible for the synthesis of ATP, the currency of energy for all organisms. When AtpD was deleted from *Mycobacterium tuberculosis*, it only produced a small amount of ATP and was in a state of energy damage [43]. Therefore, we used the molecular docking method to simulate the binding of 3-carene with important proteins (MurA, OmpW and AtpD), so as to judge the potential of the three proteins as antimicrobial targets.

It is generally believed that homologous modeling can be achieved when the sequence similarity of the template is higher than 30%. The quality of those models is evaluated by Qualitative Model Energy Analysis (QMEAN) and Global Model Quality Estimation (GMQE) in SWISS-MODEL. QMEAN Z-scores around zero indicate good agreement between the model structure and experimental structures of similar size. The resulting GMQE score is expressed as a number between 0 and 1, where higher numbers indicate higher reliability. Specific information about the models of MurA, OmpW, and AtpD is given in Table 4. The detailed interactions between the proteins and ligands are summarized in Table 5.

**Figure 7:**
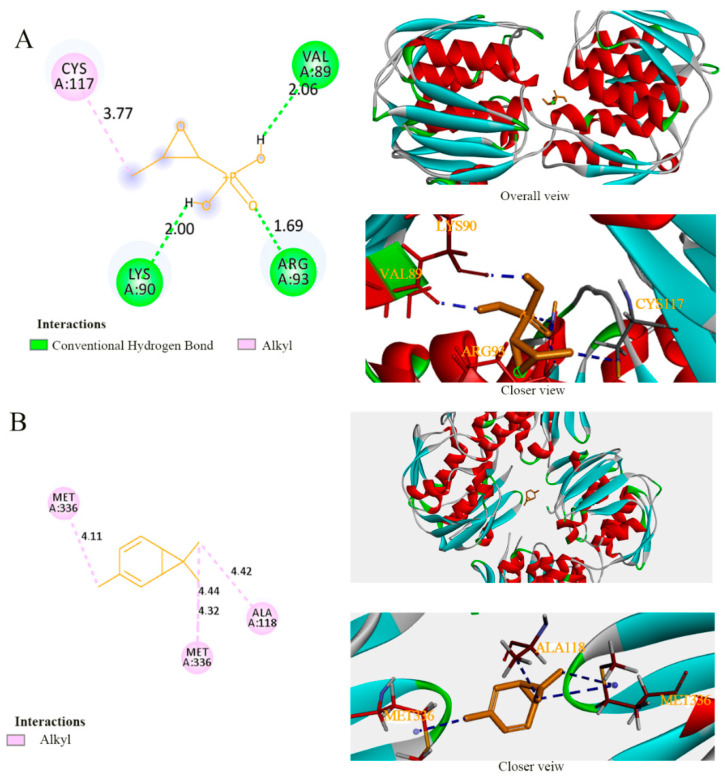
The binding details of MurA with fosfomycin (**A**) and 3-carene (**B**).

**Figure 8:**
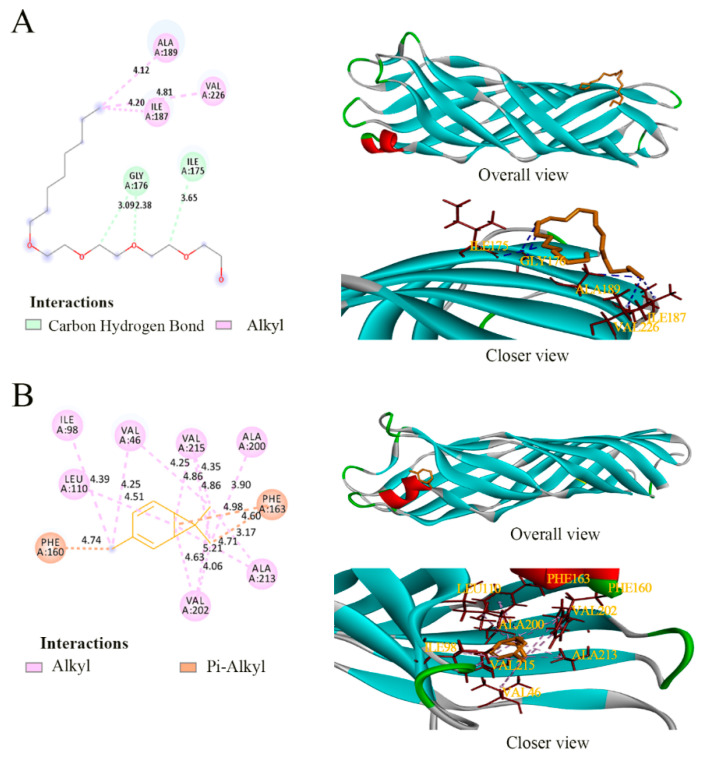
The binding details of OmpW with (hydroxyethyloxy) tri (ethyloxy) octane (**A**) and 3-carene (**B**).

**Figure 9:**
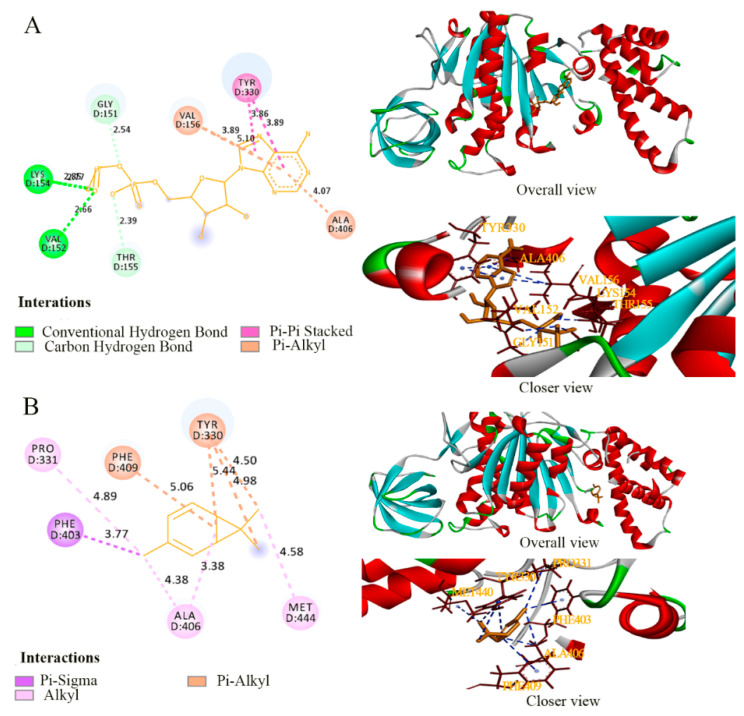
The binding details of AtpD with adenosine diphosphate (ADP) (**A**) and 3-carene (**B**).

Fosfomycin is known to selectively inhibit MurA by acting on the Cys115 residue that is reported as an active site of fosfomycin [44]. This Cys is conserved in all MurA except *M. tuberculosis*, *C. pecorum*, *P. gingivalis*, and *C. trachomatis*, and the MurA of *Acinetobacter baumannii* also encodes it (Cys116) [45], as does *P. lundensis* (Cys117). In the detailed interactions between the active sites of target proteins and ligands (Figure 7, Figure 8 and Figure 9), the binding model of fosfomycin (Figure 7A) formed alkyl–alkyl interactions with Cys117 and hydrogen bonds with Val89, Lys90, and Arg93, with a binding energy of −4.22 Kcal/mol, which was lower than that of the complex of 3-carene–MurA (−5.19 Kcal/mol). It is worth mentioning that the MurA model is a tetramer and 3-carene connected two of the monomers by linking Met336 in one chain with Met336 and Ala118 in the other chain (Figure 7B). Ompw, a transporter, has neither a substrate nor recognized inhibitor. Therefore, the original ligand from the template was used to compare the binding effect of 3-carene with OmpW (Figure 8A). The results show that the binding energy of 3-carene (−4.22 Kcal/mol) was much higher than that of the original ligand (−0.43 Kcal/mol), and 3-carene formed hydrophobic interactions with nine hydrophobic amino acid residues (Figure 8B). As the substrate of AtpD, the ADP formed two hydrogen bonds, two carbon–hydrogen bonds, pi–pi stacking, and pi–alkyl interactions with AtpD (Figure 9A), which displayed good binding energy (−6.75 Kcal/mol). However, the result of docking 3-carene with AtpD (Figure 9B) revealed that 3-carene created pi–sigma interactions with Phe403, alkyl–alkyl interactions with Pro331, Ala406, and Met444, and pi–alkyl interactions with Phe409 and Tyr330, and its binding energy was −4.93 Kcal/mol. 3-Carene and the substrate had the same two action sites on AtpD (Ala406 and Tyr330), thus meaning that 3-carene might impede the binding of ADP to the active sites, resulting in the inhibition of ATP synthesis.

Through the docking results of 3-carene with three proteins (MurA, OmpW, and AtpD), we reported that 3-carene could bind with those important proteins and produce good binding energy, which might inhibit the activity of MurA and ATP synthase, and affect the normal physiological functions of OmpW, such as controlling the import and export of substances. The results explain how 3-carene destroyed the cell structure and affected the synthesis of ATP. In addition, since 3-carene lacks hydrogen bonding receptors such as hydroxyl groups, the binding energy can be attributed to hydrophobic interaction.

### 3.8. Application of 3-Carene in Pork

**Figure 10:**
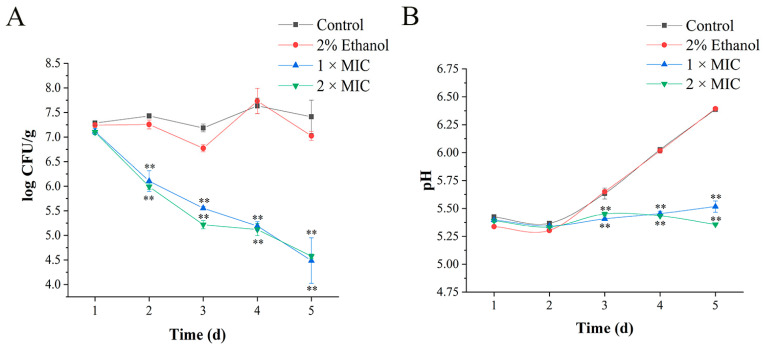
Log CFU/g of *P. lundensis* inoculated in pork samples stored at 4 °C (**A**). Changes in pH value of pork samples (**B**). The asterisks indicate statistically significant differences compared with the control (** *p* < 0.01).

During storage at 4 °C, 3-carene showed high antibacterial activity against *P. lundensis* on pork (Figure 10A). On the second day of storage, the bacteriostatic effect of 3-carene was significantly reflected and *Pseudomonas* counts in the treatment group (1 × MIC and 2 × MIC) were reduced from 7.1 to 6.1 log CFU/g, and from 7.0 to 5.9 log CFU/g, respectively. After 1 d, the number of *Pseudomonas* in the presence of 3-carene was always much smaller than that in the control group, and decreased with the increase in storage time, implying that the antibacterial activity of 3-carene was fast and durable. On day 5, the *Pseudomonas* counts in the treated group were reduced to 4.4 and 4.5 log CFU/g.

The pH value is also an important indicator of fresh pork quality. The criteria for evaluating meat freshness by pH value are as follows: the first-grade fresh meat had a pH of 5.8–6.2 [46]. According to Figure 10B, pH values in the control group and the ethanol-treated group increased during the storage period and were 6.38 and 6.39, respectively, until the last day (day 5). This was because proteins were broken down by enzymes or microorganisms into alkaline substances, such as ammonia and amine compounds, resulting in an increase in the pH of meat. On the contrary, pH values in the treated group maintained relative stability, which indicates that 3-carene prevented the increase in pH in pork. Based on these results, we concluded that when 3-carene was used as a marinade in pork at 4 °C, it reduced the population of *P. lundensis* and could maintain the freshness of pork.

## 4. Conclusions

In summary, the antibacterial mechanism of 3-carene against *P. lundensis* was demonstrated by the following aspects. Firstly, 3-carene destroyed cell morphology and the cell membrane permeability and integrity, contributing to the leakage of intracellular biomacromolecules and ions. Then, the regulation of ATPase on ions was imbalanced, and the respiratory metabolism was inhibited. We suggested that the mechanism affecting the structure and metabolism of *P. lundensis* might be the binding of 3-carene with three potential protein targets (MurA, OmpW, and AtpD) by hydrophobic interaction. When 3-carene was applied as a marinade solution in pork, the *Pseudomonas* counts were reduced significantly and pH remained relatively stable, showing an obvious preservation effect. Thus, this study confirmed that 3-carene can be effectively used for the prevention and control of *P. lundensis*, and found three potential protein targets as a foundation for the follow-up exploration of antibacterial targets.

## Figures and Tables

**Table 1:** Minimum inhibitory concentration (MIC) against *Pseudomonas lundensis*.

Bacteria	Control		The Concentration of 3-Carene (mL/L)					
*****P. lundensis*****	**Sterile Water**	**2% Ethanol**	**5**	**10**	**15**	**20**	**25**	**30**
	+++	+++	+++	+++	+++	++	−	−

“−” represents no bacteria; “++” represents a medium number of bacteria; “+++” represents a large number of colonies.

**Table 2:** Inhibition rates of three representative inhibitors and 3-carene on *P. lundensis*.

Inhibitors	Respiratory Rate R_0_ (nmol O_2_/mL/min)	Respiratory Rate after Inhibitor Treatment R_1_ (nmol O_2_/mL/min)	Inhibition Rate I_R_%
EMP Inhibitor	2.156	0.407	81.12
TCA Inhibitor	2.156	1.293	40.02
HMP Inhibitor	2.156	1.512	29.87
3-Carene	2.156	1.446	32.93

**Table 3:** The superposition rates between 3-carene and three representative inhibitors.

Inhibitors	Respiratory Rate R_2_ (nmol O_2_/mL/min)	Superposition Rate S_R_ %
3-Carene	1.446	
3-Carene and EMP inhibitor	0.237	83.6
3-Carene and TCA inhibitor	1.126	22.13
3-Carene and HMP inhibitor	1.044	27.8

**Table 4:** Scores of three proteins of *P. lundensis*.

Protein	Template Protein Data Bank (PDB) ID	Description	GMQE	QMEAN	Sequence Similarity
MurA	6CN1	UDP-N-acetylglucosamine 1-carboxyvinyl transferase	0.95	−0.08	0.58
OmpW	2X27	Outer membrane protein W	0.68	−1.02	0.45
AtpD	2V7Q	ATP synthase subunit beta	0.86	−0.63	0.52

**Table 5:** Detailed interaction between the *P. lundensis* proteins and ligands.

Protein	Ligands	Binding Energy (Kcal/mol)	Residues Binding at Ligand–Protein Complex
MurA	Fosfomycin	−4.22	Cys117 Val89 Lys90 Arg93
	3-Carene	−5.19	Met336 Ala118
OmpW	(Hydroxyethyloxy) Tri (Ethyloxy) Octane	−0.43	Gly176 Ile175 Ala189 Ile187 Val226
	3-Carene	−4.22	Ile98 Val146 Val215 Ala200 Phe163 Ala213 Val202 Leu110 Phe160
AtpD	adenosine diphosphate (ADP)	−6.75	Tyr330 Val156 Ala406 Gly151 Lys154 Val152 Thr155
	3-Carene	−4.93	Ala406 Pro331 Phe409 Tyr330 Met444 Phe403

## Data Availability

The data presented in this study are available on request from the corresponding author.
